# Sensors and Pattern Recognition Methods for Security and Industrial Applications

**DOI:** 10.3390/s22165968

**Published:** 2022-08-10

**Authors:** Michał Choraś, Rafał Kozik, Marek Pawlicki

**Affiliations:** Institute of Telecommunications and Computer Science at Bydgoszcz University of Science and Technology, PBS, 85-796 Bydgoszcz, Poland

Contemporary cyberthreats continue to evolve, powering the neverending development arms race. Critical and industrial applications are particularly sensitive to both cyber and physical attacks, placing novel security solutions in high demand.

In this Special Issue of *Sensors*, our driving idea was to invite high-quality papers that open the doors to accommodating new AI paradigms, approaches, and mechanisms in the domain of applied security. This includes pattern recognition, data analysis, and machine learning for industrial applications, including e-commerce.

The Special Issue also aimed to present novel sensors (e.g., drones, IoT) used for security, as well as innovative solutions for secure software development.

Relevant topics included: security of IoT; security of the cloud, fog, and edge networks; security and sensors in e-commerce; machine learning (shallow and deep) in security and industrial applications; sensors for security and industrial applications; innovative pattern recognition solutions; practical applications of AI in security; practical applications of AI in industrial applications; AI methods for threat prediction, detection, and mitigation anomaly detection methods; AI and machine learning for secure software; biometrics; secure AI solutions for the countering and detection of adversarial attacks. The published papers can be grouped into three types:

1. Survey papers

This Special Issue contains one survey paper entitled ‘A Systematic Review of Recommender Systems and Their Applications in Cybersecurity’ [1], by Pawlicka et al. This paper elaborates on the benefits of the possible utilisation of recommender systems in cybersecurity. It delves into the types of recommender systems, listing their particular advantages and disadvantages, bearing in mind their relevance for use in cyber applications. Having set the stage for recommender systems, the paper explores the state of the art of the use of recommender systems in cybersecurity, featuring both already-existing solutions and published ideas. To the best of our knowledge, this is the first survey accumulating the applications of recommenders in cyber. The work also performs a comprehensive survey and taxonomisation of the recommender types, presenting the most comprehensive list of the kinds of filtering, drawing from all the analysed works.

2. Threat, Intrusion, Anomaly, and Attack Detection

Cyberthreats, intrusions and attacks are a significant burden for contemporary society and various security applications rely on current research for effective applications of AI/ML paradigms in the fight against malicious cyber actors. This Special Issue contains five papers dealing with the problem of threat, intrusion, anomaly and attack detection.

Face recognition is an immensely proliferated approach to identity verification. The spread of this technology has invited malicious activities. One of the threats against face recognition is the presentation attack—using a photo or a video of a person to obtain access. In ‘Face Presentation Attack Detection Using Deep Background Subtraction’ [2], Benlamoudi et al. present a detection method against those kinds of attacks. The approach leverages a combination of background subtraction (BS) and convolutional neural network(s) (CNN), as well as a majority-vote-based ensemble of classifiers. The inclusion of the ensemble significantly improves the effectiveness of the detection method. The authors report promising results.

In ‘Presentation Attack Detection on Limited-Resource Devices Using Deep Neural Classifiers Trained on Consistent Spectrogram Fragments’ [3] the authors deal with a similar threat targeting biometric speaker verification. Kubicki et al. put forward a new method for convolutional phoneme classifier training, which results in high phoneme recognition accuracy even for heavily streamlined neural networks designed for resource-limited hardware. The method selects spectogram chunks from the central regions of phoneme articulation intervals, which maximises phoneme-related spectogram structure consistency. The effectiveness of this method is demonstrated for limited-capacity neural networks, approaching the state-of-the-art accuracy of much more complex networks.

The work of Fährmann et al. [4] focuses on the challenge of cyberattacks against industrial control systems and proposes an anomaly detection method based on long short-term memory variational autoencoders (LW-LSTM-VAE). The proposed approach is successfully evaluated in two scenarios in water purification and water distribution plants. Abhijeet Sahu et al. [5] focus on the problem of false alerts in Intrusion Detection Systems (IDS) and propose an evidence–theoretical approach leveraging Dempster–Shafer (DS) combination rules and their variants for reducing false alerts. The method utilises probability scores obtained from supervised-learning classifiers to facilitate a location-with-domain-based fusion framework as an evaluator for the detector’s performance. Plausibility, belief, pignistic, and general Bayesian theorem-based metrics are used as decision functions in the evaluation, which is performed in an emulated large-scale electric grid.

One of the major challenges of the utilisation of ML in the cybersecurity arms race is the recency and relevance of data. The work of Mihailescu et al. [6] aims to address this challenge by introducing the SIMARGL2021 Network Intrusion Detection Dataset. The dataset was collected in a real-world scenario with the authors performing and annotating the attacks. It contains 44 flow features characterising the traffic. The dataset is tested on a range of classic ML algorithms.

3. Practical AI in Industrial Applications

Different aspects of the ML pipeline find their way into a range of industrial operations. The following five papers elaborate on the practical use of Artificial Intelligence technologies in industrial applications. Zofia Długosz et al. [7] propose an improvement to the particle swarm optimisation (PSO) algorithm to be used in miniaturised devices and systems that require low energy consumption. This is obtained by a significant decrease in the computational complexity of PSO. The authors replace the mechanism responsible for random value generation with deterministic methods. The algorithm is evaluated with a range of fitness functions. The authors also put forward a hardware implementation.

Karolina Nurzynska et al. [8] present a dedicated database encompassing 27 various flight simulator devices allowing for the fast identification of objects and pinpointing them through keypoint descriptors. The authors compare the results of 12 keypoint location methods and 10 keypoint descriptors. The research contained in this paper will aid the design of a flight simulator training support system.

Mariusz Topolski [9] establishes an approach to evaluating the dimensionality of features rooted in correlation measures along with the discriminant power of features. This facilitates a more precise reduction of the dimensionality when in comparison to the Kaiser criterion. The research is done in the context of risk classification in the pharmaceutical industry. Having performed a series of experiments, the author also identifies a set of cybersecurity-related characteristics that influence the risk assessment of chemical hazards along with the concentration of volatile substances. Teresa Pamuła et al. [10] leverage deep learning for the detection of obstacles at rail-level crossings. This is achieved by processing video data for the measurement of the state of the crossing region. The proposed approach is evaluated on real-life video surveillance data gathered in Poland in different weather conditions, seasons, and light conditions. The method reaches 98% recall and could be used to aid train control systems.

In ‘Differential Diagnosis of Cysts and Granulomas Supported by Texture Analysis of Intraoral Radiographs’ [11], Elżbieta Pociask et al. consider whether texture analysis can be applied in the task of distinguishing between radiographic images of lytic lesions. The research used over 10,000 features to form logistic regression models on data coming from 62 patients and a historical study. The work positively verified the possibility of distinguishing between cysts and granulomas with the use of textural analysis regardless of lossy compression of the images and unpreserved scale of the images.

The selected authors of the contributions presented on 12th International Conference on Computer Recognition Systems (CORES) and the 12th International Conference on Image Processing and Communications (IP&C), held jointly with the 22nd International Conference on Advanced Computer Systems (ACS), were invited to submit extended versions of their original papers for this Special Issue. Our multi-conference took place in June 2021. This Special Issue was, of course, open to submissions outside of the conference, and many authors used this opportunity. We plan to organise another edition of our conferences (IP&C and CORES) in June 2023 in Wrocław Poland, and we hope to meet in person there.

We would like to express our profound appreciation for the authors and reviewers who made this Special Issue possible.

In the time it took to complete this Special Issue, our reviewers and authors have exerted tremendous effort into letting it come to life and flourish. All the accepted papers underwent at least two rounds of revisions, always with at least the reviewers (selected by the journal, not by the Guest Editors of this Special Issue). In the back-and-forth between the reviewers and the authors, we strove to ensure that the papers could reach excellence in every aspect. This high number of revisions posed a tremendous workload on everyone involved—but we believe the result was worth it.
